# The Variety of Applications of *Hermetia illucens* in Industrial and Agricultural Areas—Review

**DOI:** 10.3390/biology12010025

**Published:** 2022-12-22

**Authors:** Monika Kaczor, Piotr Bulak, Kinga Proc-Pietrycha, Marina Kirichenko-Babko, Andrzej Bieganowski

**Affiliations:** 1 Institute of Agrophysics, Polish Academy of Sciences, Doświadczalna 4, 20-290 Lublin, Poland; 2 Schmalhausen Institute of Zoology, National Academy of Sciences of Ukraine, B. Khmelnitsky 15, 01030 Kyiv, Ukraine

**Keywords:** black soldier fly, revalorization, circular economy, waste management, biogas, biodiesel, entomoremediation, frass, AMP, chitin

## Abstract

**Simple Summary:**

Human population growth contributes to a negative impact on the environment. In order to protect and restore nature, finding solutions and technologies in the industrial and agricultural fields that simultaneously recycle organic waste biomass, revalorize and recover nutrients and natural compounds is continuously important. The production of the insect *Hermetia illucens* (Diptera: Stratiomyidae, Linnaeus, 1758) fits well within the framework of green policy. *H. illucens* larvae fed on various biomass. The redirection of leftovers from fruit and vegetable or food processing to feed the larvae allows to produce insect proteins and fat, which can be further used in the production of animal feed. Besides, the larvae are also able to feed on manure, biogas sludge, and municipal sewage sludge, which decreases its weight and thus offers entomoremediation of the waste. Insect frass is use as an organic fertilizer. Fats and insect biomass are suitable for biodiesel production and biogas generation. From insect exoskeletons, chitin and chitosan are extracted. Thus, insect production seems to create new and unique opportunities for the environment, people, and animal nutrition, and the large and growing number of publications on *H. illucens* puts it in the center of interest of various research communities.

**Abstract:**

*Hermetia illucens* (Diptera: Stratiomyidae, Linnaeus, 1978), commonly known as the black soldier fly (BSF), is a saprophytic insect, which in recent years has attracted significant attention from both the scientific community and industry. The unrestrained appetite of the larvae, the ability to forage on various organic waste, and the rapid growth and low environmental impact of its breeding has made it one of the insect species bred on an industrial scale, in the hope of producing fodder or other ingredients for various animals. The variety of research related to this insect has shown that feed production is not the only benefit of its use. *H. illucens* has many features and properties that could be of interest from the point of view of many other industries. Biomass utilization, chitin and chitosan source, biogas, and biodiesel production, entomoremediation, the antimicrobial properties of its peptides, and the fertilizer potential of its wastes, are just some of its potential uses. This review brings together the work of four years of study into *H. illucens*. It summarizes the current state of knowledge and introduces the characteristics of this insect that may be helpful in managing its breeding, as well as its use in agro-industrial fields. Knowledge gaps and under-studied areas were also highlighted, which could help identify future research directions.

## 1. Introduction

Insects are one of the most diverse classes within the animal division. They occur in all climatic zones and in terrestrial and aquatic ecosystems [1]. From an anthropocentric point of view, around 5.5 million insect species worldwide [2] can be categorized as being either useful or harmful to human interests [3]. One of the beneficial insects is *Hermetia illucens* (Linnaeus, 1758), often referred to as the black soldier fly (BSF) [4].

*H. illucens* belongs to the order of Diptera, in the family of Stratiomyidae. Their indigenous regions are North and South America, but nowadays this fly has also been observed on other continents in tropical, subtropical, and temperate zones [5]. The fly belongs to the holometabolous insects and its developmental cycle consists of several stages: eggs, larvae, prepupae, pupae, and adult [6]. The larvae of *H. illucens* are saprophagous, which means that they feed on various rotten organic matter. It is the only stage in the developmental cycle that can consume food. Adult *H. illucens* flies are able to intake water but it derives most of energy from reserves accumulated in the larval stage. Their only goal is to reproduce. Studies have shown that flies with access to protein (a mixture of sugar, milk protein, and bacteriological peptone) can live about 5 days longer and lay up to three times more eggs [7]. 

**Figure 1:**
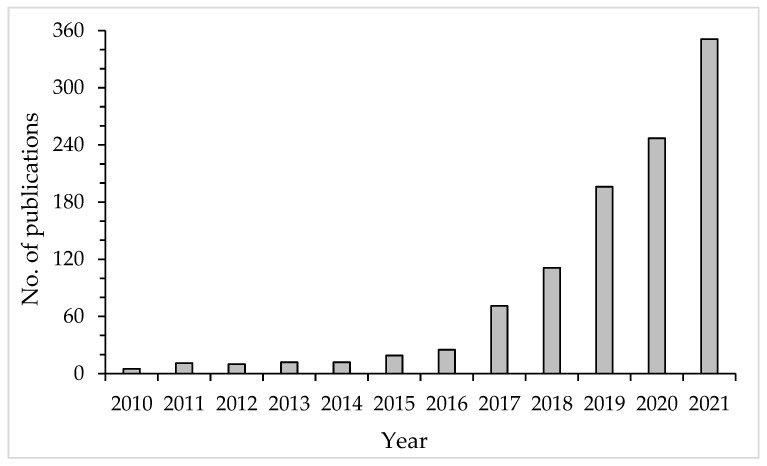
The dynamics of publication growth about *H. illucens*. Information from the Web of Sciences database, May 2022. For the search a combination of keywords was used: “*Hermetia illucens*” or “*H. illucens*” or “black soldier fly” and these words were entered into “all fields” in the form of core collection.

Considering the significant amount of research devoted to insects, it should be stated that *H. illucens* was rarely studied in the past. According to the Web of Science, up until 2009, this fly was the object of investigations on just 40 occasions. Only in the last 5 years has there been a significant increase in the interest in *H. illucens* (Figure 1).

Nevertheless, until now, very few reviews describing the collective aspects of its use in industrial and agricultural applications have appeared [4,8]. The aim of this study is to gather the latest information from studies which have appeared since 2018, fill in certain information gaps, and present a broader spectrum of the industrial and agricultural applications of *H. illucens*. Moreover, on the basis of the analysis, we propose research or application areas with future potential in both scientific and industrial fields.

## 2. Applications of *H. illucens*

### 2.1. Bioconversion of Waste Biomass

Undoubtedly, interest in *H. illucens* has increased due to its ability to develop properly and increase its body mass, as well as its high survival rate when consuming different types of food. The insect’s diet may include various types of organic waste, agronomic waste ranging from crop residues, such as corn straw [9], seasonal agri-food residues [10], animal manure [11,12,13], municipal waste in the form of sewage sludge [14,15], as well as food waste, i.e., vegetable and fruit waste [16,17], or restaurant waste [18]. The performance of *H. illucens* larvae resemble fast and efficient bioconverters of waste biomass. Their uses, as well as those of other saprophytic insects, may be very important for bioconversion technology, due to the growth of the global population and its demand for food. This, unfortunately, means more food being discarded or becoming outdated, which is either wasted or ends up in landfill, occupying more and more land areas and contributing to uncontrolled greenhouse gas emissions. The cultivation of *H. illucens* larvae could be a possible means of utilizing waste biomass, while simultaneously obtaining larval biomass that could be a source of nutrients, such as proteins or fats [19]. In this context, the process may also be referred to as the revalorization of waste biomass, as the waste may become feed for the insect.

A compilation of data on the crude protein, the crude fat content of larvae, as well as bioconversion factors, and the dry matter reduction values of different substrates, are presented in a review article by [20]. Depending on the substrates, the protein content of the larvae ranged from 30.7–53.0% dry mass (DM), with the highest value obtained in brewery by-products. As regards larval fat, the content ranges from 8.1–47.4% DM, with the highest value obtained in fruit waste [20]. The highest biomass reduction values were obtained in substrates, such as municipal organic waste (68.0% DM) [21], chicken manure (61.7% DM) [22], a mixture of food waste and human excreta (68.4–68.8% DM) [23], a mixture of slaughterhouse waste, fruit, and vegetable waste (61.1% DM), and primary sludge (63.3% DM) [14].

Lalander et al. [14] conducted a study which evaluated the development of *H. illucens* larvae and determined the factors affecting the efficiency of bioconversion. For this purpose, several different substrates were tested: poultry feed, dog feed, food waste, fruit and vegetables, abattoir waste, fruit and vegetable waste, poultry manure, human feces, primary sludge, undigested sludge, and digested sludge. *H. illucens* larvae reared on these media contained protein in their biomass at 39.1–44.2% DM, with the highest value obtained in abattoir waste. The results of this study showed that the volatile solids (VS) content and protein content of the feed medium were the main factors affecting the timing and growth of larvae [14].

Liu et al. [24] found that a high presence of carbon component with poor digestibility, such as lignin and cellulose, had a strong negative influence on *H. illucens* larvae development. Ramzy et al. [25] also came to this conclusion by conducting an experiment with different doses of olive pomace residue, which was rich in lignin. The variant, which contained 39.54% of lignin fraction, resulted in larvae with a body weight of approximately 220 mg, which was 1.3 times lower than the control. Simultaneously, a lower larval survival rate of 75.5% was recorded, which was 1.2 times lower than the control [25].

Extensive analyses of the utilization rates of various substrates by *H. illucens* larvae have been published in recent years, among which are the following: [6,26,27,28].

### 2.2. Animal Feed

The use of *H. illucens* to feed a wide variety of animals is one of the most studied practical aspects of the use of this insect. Presently, there are review articles which focus exclusively on this use [29,30,31].

There are several possible ways that *H. illucens* can be used as a feed for animals. Whole larvae and pupae can be given to animals such as chickens [32,33,34], reptiles [35], fish/aquatic animals [36,37,38], pigs [39,40] or even dogs [41]. The direct use of whole and live *H. illucens* was certainly the easiest and the most practical and inexpensive way of administering food to animals [34]. Whole insects can also be dried and ground and, in this form, constitute a feed additive [39].

The nutritional components of the insect, such as fat and proteins, can also be extracted and used as feed additives or to formulate new feeds. *H. illucens* is characterized by an appropriate amino acid composition both for animal and human consumption [42]. If insect fat is used, this can be a substitute for soybean oil [43]. It is also possible that appropriate modifications of the *H. illucens* larvae diet can improve the quality of the larvae fat, e.g., enrichment with fish residues results in an increase in the omega-3 fatty acid content in the larvae [44].

In the case of obtaining protein meal, there are three types: defatted [41], partially defatted [45] or full fat [40]. In this case, the choice of the correct type of feed should be guided by the preferences and nutritional requirements of the particular animal. This approach requires the industrial processing of raw insects, which incurs higher costs but has the advantage of creating new feed formulas, ideally suited to the nutritional requirements of a given animal.

Research has found that defatting is important when producing powdered feed from the insect, otherwise difficulties could occur during the pulverizing process, due to the high content of fatty components in the bodies of *H. illucens* [46]. Kim et al. [46] proposed two methods to remove the fat from dried and ground larvae: through the use of hot water, which reduced the fat level from the initial 30 to 16%, and the more efficient, supercritical carbon dioxide extraction method, which eventually reduced the fat level to 4.5%. This process was conducted for 6 h under an increased pressure of 350 bar and a flow rate of 26 L CO_2_·h^−1^ [46].

The variety of animals that have been fed by supplements produced from *H. illucens* is considerable (Table 1). *H. illucens*, as a feed, mostly had a positive effect on various animal organisms [47,48], however, in some cases, researchers reported a negative influence, manifesting, for example, in a slightly reduced body weight or the reduced digestibility of proteins and lipids [37,49]. This indicated that *H. illucens* meal is not optimal nutrient source for all animals. For instance, [37] concluded that *H. illucens* should not make up more than 17% FW of the feed composition for fish *Argyrosomus regius*, so as not to cause undesirable effects [37] (Table 1).

In addition to providing nutrients, studies have shown that feed from *H. illucens* also contained antimicrobial and antioxidant properties, as well as beneficial influence on the intestinal tract of animals (Table 1). Dabbou et al. [43] investigated the antimicrobial potential of the fat of *H. illucens*, as well as the use of insect fat as a replacement for soybean oil added to rabbit feed, at a value of 1.5%. Subsequently, an investigation was conducted to evaluate the way in which this change in diet affects the intestines of the animals.

The antimicrobial activity of the fat of *H. illucens* was tested on bacterial strains, which represent serious pathogens and are capable of causing diseases or poisoning of the digestive system. The results showed that the fat of *H. illucens* had no effect on *Salmonella* bacterial strains (*S. tiphymurium*, *S. enteritidis*). In the case of *Yersinia enterocolitica*, *Pasteurella multocida,* and *Listeria monocytogene*, bacteriostatic effects were noted [43].

Regarding the effect of the fat of *H. illucens* on the intestinal function, compared to the control, a difference was observed in the amount of volatile fatty acid (VFA) production in the cecum, which was recorded as 85.3 mmol·L^−1^ and was 1.2 times higher than the control; this could be the result of changes made in the intestinal microbiome of the rabbits. The addition of the fat of *H. illucens* increased the amount of *Ruminococcus* bacteria, which produce short chain fatty acids and have a positive effect on the microbiome of herbivores [43].

Yu et at. [39] investigated changes in the gut microbiome and the bacterial metabolites produced in female finishing pigs (crossbred Duroc, Landrace, and Large White), whose diets included 4% dried and crushed *H. illucens* prepupae. Pigs fed with a diet of *H. illucens* showed a higher concentration of beneficial *Lactobacillus* and *Pseudobutyrivibrio*, *Roseburia* and *Faecalibacterium*—butyrate-producing bacteria in their intestines, with a reduced concentration of *Streptococcus* bacteria. In addition, the inclusion of *H. illucens* in the pig diet caused the expression of pro-inflammatory genes to decrease, while the expression of anti-inflammatory genes increased [39].

In another animal study, Lei et al. [41] proved the antioxidant and anti-inflammatory properties of *H. illucens* supplementation, by studying how it affected beagle dogs. A 1% or 2% addition of defatted *H. illucens* larvae meal sample was added to the dogs’ diet which consisted of cereal grains. In addition, at the end of the 6 week experiment, all dogs were injected with *Escherichia coli* lipopolysaccharide (LPS; 100 μg·kg^−1^ body weight) to induce an inflammatory response. An analysis of serum parameters showed that the only difference between the control and the *H. illucens* larvae meal samples was the albumin concentration. With a 2% *H. illucens* larvae addition, the albumin concentration was 1.3 times higher than in the control. There was also an increase in dry matter (75.21%) and crude protein (78.51%) digestibility by 1.05 and 1.07 times, respectively, compared to the control. In a blood profile study after *E. coli* LPS injection, there was a lower tumor necrosis factor-α concentration in the blood of the dogs, which were fed the 2% *H. illucens* larvae feed; this was 1.82 times lower than the control after 6 h. Moreover, an increase in the concentration of glutathione peroxidase, which has an antioxidant function, was observed. Its activity was 53.05 nmol·min^−1^·mL^−1^, which was 1.26 higher than the control. In the case of superoxide dismutase, an increase in its concentration was observed in the dogs fed with *H. illucens* larvae meal 3 h after injection, with concentrations subsequently decreasing. The results maintained in this study indicate the positive effect of the addition of *H. illucens* to beagle dog food, demonstrating health-promoting activities [41].

In a study involving the replacement of 100% soybean oil with fat from *H. illucens* larvae in the diets of male broilers, Cullere et al. [51] recorded no change in the nutritional composition and sensory profile. However, a difference could be observed in the fatty acid profile, in which the amount of saturated fatty acid (SFA) increased, while the amount of polyunsaturated fatty acids (PUFA) decreased. In chicken breast, SFA increased 1.6 times more than the controls and was 45.8% of the total fatty acid methyl esters (FAME). PUFA concentration decreased 1.6 times more than the control and was recorded at 23.4%. In the leg meat, on the other hand, the SFA was 1.7 times greater than the control, being 44.2%, and the amount of PUFA decreased 1.6 times compared to the control and was recorded at 24.3% [51]. One of the reasons could be that the lipid fraction of *H. illucens* is deficient in PUFA, which are essential components for the correct development of, e.g., aquaculture animals [52,53]. An increase in the fat content of these *H. illucens* larvae is therefore a positive direction in which further research should be conducted. An interesting approach was proposed by [54], who reared *H. illucens* larvae on a diet enriched with *Schizochytrium* algae, which are rich in omega-3 PUFA. The authors found that the *H. illucens* larvae bioaccumulated this. This treatment increased the amount of PUFA in the larval biomass from 13.6% in the control to a level of 22.2% [54].

In terms of feeding, mineral composition is key. As with the basal composition of *H. illucens* larvae, the mineral composition depends on the substrate in which the larvae were bred. A review by Barragan-Fonseca et al. [55] compares the mineral values in the larvae fed with chicken feed (CF) and with poultry and swine manure. The element that was found in the greatest amounts in the body of the larvae was Ca. Its content in the larvae fed with CF was 31.4 g·kg^−1^ DM, while the larvae on both manures contained 50–78 g·kg^−1^ DM. The higher concentration of macroelements such as P, Mg, Na, and K, was obtained in larvae fed with CF, and their values were 12.8, 7.9, 2.7, and 19.6 g·kg^−1^ DM, respectively. A higher content of the microelements Fe, Zn, Cu and Mn were found in larvae fed on manures, and were recorded as 100–1400 µg·g^−1^ DM, 100–300 µg·g^−1^ DM, 10–30 µg·g^−1^ DM, and 200–600 µg·g^−1^ DM, respectively [55].

In a study by Surendra et al. [20], there was a compilation of the mineral compositions of *H. illucens* prepupae, reared on different types of organic wastes. The richest in mineral pupae were those fed with digestate (following an anaerobic fermentation of the mixture of vegetables) or brown algae (*Ascophyllum nodosum*). With regard to the digestate, the highest amounts were found for Ca—66.15 g·kg^−1^ DM, Fe—6.75 g·kg^−1^ DM, Mn - 0.38 g·kg^−1^ DM, and S—0.31 g·kg^−1^ DM. In the case of brown algae, the highest amounts of K—21.30 g·kg^−1^ DM, Mg—6.20 g·kg^−1^ DM, Na—12.30 g·kg^−1^ DM, P—12.30 g·kg^−1^ DM, and Zn—0.15 g·kg^−1^ DM were obtained [20]. The substrate supplement given to the larvae to increase the micro- and macronutrient could be fruits. Romano et al. [56] studied the mineral value of larvae fed with bananas (three variants: banana peels, banana fruit, mixture of both (1:1 *w*:*w*)) and orange fruit (three variants: orange peels, orange fruit, mixture of both (1:1 *w*:*w*)). The Ca content in the larvae fed with banana mixture was 579 µg·g^−1^ DM. However, most of the minerals in the larvae were present when they were fed solely banana peels. In the case of the macronutrients, the P, K, and Mg contents were 13.27, 65.28, and 2.38 µg·g^−1^ DM, respectively. Regarding the micronutrients for Na, Mn, Zn, and Cu, the values were 77.4, 11.3, 17.8, 4.6 µg·g^−1^ DM. Only in larvae fed with the orange mixture was the presence of Ni recorded and it was 0.6 µg·g^−1^ DM [56].

An important context of the research is the study of *H. illucens* for elements that may be harmful to the health of the organisms. These insects have the ability to bioaccumulate elements, and attention should be paid to the composition of the substrate on which they are fed. More information on the bioaccumulation of elements and other contaminants is discussed in this review in Section 2.7.

The effect of feeding animals with *H. illucens* on properties and characteristics, such as fat profiles, meat quality, and heavy metal content in meat, was also studied. A study conducted on broilers showed that replacing 25% soybean meal with microwave-dried and press-defatted *H. illucens* meal did not harm the health of the animal or the meat quality [57]. The body weight of a chicken fed on a diet consisting of a 25% addition of *H. illucens* was 1.76 kg (live weight) compared to 1.80 kg among the control on soybean meal. However, a higher percentage addition (50%) of *H. illucens* to the broiler diet resulted in a 1.2-fold reduction in body weight gain compared to the control [57].

There are also studies which indicated that the addition of *H. illucens* to animal feed yielded neither positive effects [36,38] nor negative effects on, e.g., the weight gain of animals [37,49,58]. The negative effects should be investigated further to understand why, in some cases, *H. illucens* is not a good choice for animal nutrition. However, in the case of neither positive nor negative effects on animal growth, the addition of *H. illucens* could still, in some cases, have an economic justification and could prove to be a cheaper counterpart by comparison with other more commonly used nutritional supplements.

### 2.3. Chitin and Chitosan

#### 2.3.1. Chitin

Chitin is a carbohydrate biopolymer of N-acetyl-D-glucosamine and D-glucosamine units [59]. It occurs in various living organisms, such as fungi [60], mollusks [61] and insects [62] and can have different physicochemical properties depending on its origins and internal arrangement of fibers [63]. Those arrangements differentiate chitin to α-, β-, and γ-chitin form [63]. Chitin present in *H. illucens* occurs in the α configuration [64]. The α-chitin molecule has an antiparallel arrangement of carbohydrate chains and is the most thermodynamically stable form of chitin [63]. Chitin content varies between *H. illucens* developmental stages, but unsurprisingly the highest amounts are found in the puparium (Table 2) [65]. It was also found that the chitin content in the internal organs of *H. illucens* is about 20% of the total chitin content [64,66].

To obtain this compound, the standard and most commonly used method is the sequential removal of the remaining larval fractions. Firstly, the sample is demineralized with 1 M HCl, followed by a deproteinization process in the presence of 1 M NaOH. Chitin from *H. illucens* larvae is strongly bound with melanin, therefore a depigmentation step is necessary. Chemicals such as KMnO_4_ [64], H_2_O_2_ [69], and NaClO [65], can be used to remove the pigments. However, this standard approach is an environmentally unfriendly way of obtaining chitin, because it produces large amounts of highly acidic or alkaline wastewater and also uses a significant quantity of water [59].

New efficient methods for chitin extraction, such as deep eutectic solvents (DES), have been developed. They are a combination of two components that act as a hydrogen bond donor and a hydrogen bond acceptor. In addition, these solvents have low toxicity and can be reused several times [71]. Natural deep eutectic solvents (NADES), such as choline chloride, betaine, urea, glycerol, DL-lactic acid, oxalic acid, and n-butyric acid, were used to isolate the chitin from the defatted *H. illucens* prepupae [72]. The best results were obtained for choline chloride-urea and betaine-urea variants at 50 °C; the chitin yield was 26.0% and 26.7%, respectively, with a purity of 88.4% and 83.3% [72].

Another method is the extraction of chitin from dried and shredded *H. illucens* puparia by microbial fermentation using *Bacillus licheniformis* A6 [59]. For depigmentation, the chitin pellet was heated in the solution of 0.1% SDS and 0.5% NaHCO_3_ and then left in 30% H_2_O_2_The chitin content obtained by this method was 12.4% [59].

Chitin extraction studies involving organic acids, ethanol, and a bacterial enzyme (*B. licheniformis* protease) were also performed [73]. Using these reagents, four different methods of extracting chitin from the dried puparia of *H. illucens* were tested. Among these isolation methods, *B. licheniformis* protease was the most effective, followed by degreasing with organic solvents. This removed 33.3% of the protein content and 38.5% of the lipid content compared to the untreated puparia. The percentage yield of chitin for all above-mentioned methods was 65.4–72.4%. The authors conclude that such high yields may be indicative of contamination [73]. Unfortunately, these methods, although environmentally friendly, do not yield good quality chitin [73].

Hahn et al. [74] conducted extensive research into optimizing the process of obtaining and purifying chitin from *H. illucens* puparia. They tested various chemicals for the demineralization, deproteinization and depigmentation steps. The optimum demineralization result, with an efficiency of around 90%, was obtained for 0.5 M formic acid, while a 96% of protein removal was achieved with 1.25 M NaOH. The results showed that the best method for chitin depigmentation was the use of 6% NaOCl (around 66% efficiency). Similarly, by using a mixture of 5% H_2_O_2_ + 1.8% NaOH + 0.2% MgSO_4_ an efficiency value of approximately 62.2% was achieved. As the authors point out, this can minimize the use of chlorine [74].

A method that requires fewer quantities of harsh chemical solutions to isolate chitin is the use of acid detergent fiber (ADF) and acid detergent lignin (ADL) methodology. The defatted sample was added to 0.5 M H_2_SO_4_ with CTAB (20 g·L^−1^), then boiled in a reflux condenser. The ADF content was calculated from the dry matter loss. The remaining filtrate was added to 12 M H_2_SO_4_, mixed, percolated and after 30 min was mixed again with 25 mL H_2_SO_4_ for 3 h, then washed and dried [68]. The chitin content obtained by this method was 21.19%, which was 1.2 times lower for puparia, compared to the conventional method (with HCl and NaOH). However, in the case of flakes obtained after oil extraction chitin content was higher by 1.3 times (26.78%). For flies, both methods gave comparable results (7.75–7.94%) [68].

Chitin may be used in environmental applications by utilizing its molecules as a sorbent for heavy metals, dyes, or air pollutants, as well as in energy applications including biosensors, and as cells used for energy production (see review of [75]). Chitin is a polymer that may also find its place in the production of biomedical materials, such as surgical sutures, wound patches or even materials used for tissue engineering [76]. One of the applications of chitin is to obtain chitosan, which will be discussed in more detail.

#### 2.3.2. Chitosan

Chitosan is a derivative of chitin, formed by its deacetylation. Thanks to its free amino groups and decreased molecular weight, it is more reactive and soluble in acidic conditions [77]. To characterize and distinguish a polymer molecule as a chitosan, its deacetylation degree (DD) must be higher than 50% [78].

A common deacetylation method is the use of 50% (*w*:*w*) NaOH, in which chitin is left for 0.5 h at room temperature, then heated to 100 °C for 2 h, followed by cooling. The resulting suspension is washed with water to neutralize the pH [79].

New methods for chitosan deacetylation were reported by [80], described as heterogeneous and homogeneous. The heterogeneous methods were conducted in a high-pressure autoclave and involved the addition of deproteinized *H. illucens* larval exoskeletons (LE) in 12 M NaOH. The headspace of the reactor was filled with nitrogen and the pressure was brought to 3 bar at 120–140 °C. After centrifugation the pellets were washed with distilled water and then added to 1% acetic acid, mixed and centrifuged again. The supernatant was brought to pH 8 and left to precipitate the chitosan [80].

The homogeneous method involved incubating a sample of deproteinized LE under cooled conditions (4 °C) in 10 M NaOH for 12 h. Then, desalinated ice was added to facilitate chitin deacetylation and stirred until the ice melted. In order to avoid the oxidation of the chitosan, the headspace was filled with nitrogen. After thawing, the solution was brought to a pH 8, incubated at 4 °C and then subjected to vacuum filtration [80]. Heterogenous conditions allowed to obtain a DD of 43–72% and a chitosan deacetylation yield of 22–47%. In comparison, the method under homogeneous conditions recorded 38% for DD and 30% for deacetylation yield [80].

Lee et al. [81] studied the structure of chitosan from *H. illucens* larvae by spectroscopic methods, as well as the antioxidant activity of its hydrolysate, made with the use of chitosanase. The obtained chitosan had a DD of 85.7% and molecular weight of 680 kDa. A test of the hydrolysate, by analyzing the radical scavenging activity of ABTS, confirmed the antioxidant properties of the sample [81].

Recent research has focused on testing the antimicrobial properties of chitosan derived from *H. illucens*. Guarnieri et al. [82] tested the antimicrobial properties of bleached and unbleached chitosan from the larvae, as well as the insect reared residues, i.e., exuviae and dead flies on the Gram-positive *Micrococcus flavus* and the Gram-negative *E. coli* [82]. All chitosan variants showed inhibitory effects regarding the growth of these bacteria. A minimum inhibitory concentration (MIC) was determined, which ranged from 0.15–0.3 mg·mL^−1^ depending on the origin of the chitosan [82].

Alghuthaymi et al. [83] produced nanocomposite made from *H. illucens* larvae chitosan and gum Arabic, to which a conjugate with eugenol and biosynthetic selenium nanoparticles was attached. It was verified for its bactericidal activity in relation to *E. coli* and *S. aureus*. The activity was greater than that of the ampicillin by approximately 1.2 times. MICs of 15.0 µg·mL^−1^ for *E. coli* and 20.0 µg·mL^−1^ for *S. aureus* were determined [83].

Several reviews described the applications of chitosan, including its antimicrobial properties [84], characteristic features used to build nanomaterials [85], use in drug delivery [86], the food industry [87], cosmetics, medical fields [88] and even environmental protection, e.g., wastewater treatment [89]. These provide the basis for future research into the use of chitosan derived from *H. illucens*.

### 2.4. Antimicrobial Properties

*H. illucens*, whose larvae live in substrates also inhabited by diverse microflora, had to develop antimicrobial mechanisms [90]. Many research studies have shown that these resistance mechanisms are quite strong. For instance, [91] used human feces as a substrate in which to grow *H. illucens* larvae and showed a 6 log_10_ reduction (99.9999%) in *Salmonella* spp. In the leftovers from the initial amount of this bacterium. The same authors, in a continuous-flow reactor study, fed larvae an artificial, pathogen-contaminated feed mix. They reported a practically complete removal of *Salmonella* spp. From approximately 10^7^ CFU·g^−1^ to below detection levels [92]. This antimicrobial effect of living larvae was related to its secretions, as well as the microflora inhabiting their entrails. Kawasaki et al. [93] found a reduction of *E. coli* in the *H. illucens* larvae frass from organic household waste.

More recent studies have investigated the effect of the addition of *H. illucens* larvae on bacterial levels in different types of manure and sewage sludge. The changes that were recorded after the application of *H. illucens* showed a reduction of pathogenic bacteria by 90–92% in chicken and cow manure, and by 86–88% in pig manure and sewage sludge. The pathogenic bacteria studied belonged mainly to the genera *Actinobacteria*, *Bacteroidetes*, *Firmicutes,* and *Proteobacteria* [94].

The common way to test the antimicrobial activity of *H. illucens* is to prepare methanol extracts from the insect. Choi et al. [90] showed that methanol extracts inhibit the Gram-negative bacteria *Klebsiella pneumoniae*, *Neisseria gonorrhoeae,* and *Shigella sonnei*, with a MIC of the extracts of 44.74 mg·mL^−1^, 43.98 mg·mL^−1^, and 43.96 mg·mL^−1^, respectively [90].

The acidic water-methanol extraction (AWME) method was used to obtain an larvae extract that was used for the first time against phytopathogenic bacteria: *Pantoea agglomerans*, *Xanthomonas campestris*, *Pectobacterium carotovorum* subsp. *carotovorum*, *Pectobacterium atrosepticum,* and *Dickeya solani* [95]. The turbidimetric assay showed that for all tested pathogens the MIC was 0.78 mg·mL^−1^ and minimum bactericidal concentration (MBC) was in the range of 0.78–1.56 mg·mL^−1^ [95].

AMWE was also tested against various strains of *K. pneumoniae*. The results showed that its MIC and MBC of 250 mg·mL^−1^ showed antimicrobial activity. In addition, bacterial membrane permeability was found to be AMWE dose-dependent, and the bacteria did not gain resistance during prolonged use of the *H. illucens* extract [96].

One of the immune responses of insects attacked by pathogens is the production of antimicrobial peptides (AMPs), from which preparations can be made to eliminate or inhibit microorganisms. To increase the production of AMPs, the immunization of the larvae of *H. illucens* can be carried out with pathogenic [97], non-pathogenic or even probiotic bacterial strains [98]. These authors demonstrated the antimicrobial activity of isolated AMPs against Gram-positive pathogens such as methicillin-resistant *S. aureus* (MRSA) 40881, *S. aureus* 12256, *S. epidermidis* and *B. subtilis*, Gram-negative *K. pneumoniae* (ATCC 13883) and *Shigella dysenteriae* (ATCC 9750). A more in-depth review of the AMPs of *H. illucens* origin, including older articles, was compiled by [99].

The process of immunization of *H. illucens* larvae, with probiotic bacteria, was studied by [100] using species of the *Lactobacillus* family (*Lactobacillus acidophilus*, *L. brevis*, *L. casei*, *L. fermentum*, *L. delbrueckii*). The highest antimicrobial activity was recorded using *L. casei* as an immunizer. They obtained hemolymph extracts of *H. illucens* from the supernatant, formed after suspending the powdered larvae in 20% acetic acid dissolved in sterile water. It exhibited inhibitory activity in relation to the bacteria of the genus *Salmonella* (*S. pullorum*, *S. typhimurium* and *S. enteritidis*), *S. aureus* and *E. coli* with a MIC in the range of 100~200 μg·100 μL^−1^, without inducing a cytotoxicity effect in animal cells as verified on CaCo-2 and L929 cell lines. In addition, the stability of the extract was found to be maintained at an elevated temperature (60–90 °C) for 24 h in a wide pH range of 2–11 [100].

Lee et al. [101] used *L. casei* immunized larvae extract. The MIC concentrations for *Enterococcus faecalis*, *Streptococcus mutans* and *Candida vaginitis* were 200, 500 and 1000 μg·100 μL^−1^, respectively. For MRSA and multidrug-resistant *Pseudomonas aeruginosa*, the MIC was ~300 μg·100 μL^−1^. Due to the good antimicrobial performance of the *H. illucens* extract, a mass injection system was developed to accelerate and automate the immunization process [101].

Shin et al. [102] screened the cDNA libraries from the fat bodies of *H. illucens* and noted a novel attacin-like peptide. Using a prokaryotic expression system, they produced a recombinant protein that was found to have properties resistant to *E. coli*, as well as MRSA [102]. Di Somma et al. [103] also used bioinformatics tools to target a peptide from the defensin family (C-15867) identified in *H. illucens* and carried out production of the peptide using gene expression by *E. coli*. The produced peptide showed inhibitory activity for *E.coli*, *S. aureus* and *Staphylococcus epidermidis* strains at an MIC of about 62 µM [103]. A recombinant *E. coli* expression system was also used to clone three AMPs (Hidefensin-1, Hidiptericin-1 and HiCG13551) produced by *H. illucens* immunized with *S. aureus*, *Pseudomonas aeruginosa* and *B. bombyseptieus*. The obtained products showed activity against *S. pneumoniae*, *E. coli* and *S. aureus* [104]. Proteomic and lipidomic analysis of the *H. illucens* larvae revealed the content of B3MI87 protein belonging to the hexamerin family, which registered activity similar to enzyme phenoloxidase-2, as well as resistance to some fungi and G-positive bacteria [105].

*H. illucens* was the first example of an insect from which a peptide with inhibitory activity against *Helicobacter pylori* was isolated [106]. Isolation has been carried out from the larvae preinoculated with *E. coli* (ATCC 25922). These peptides exhibited similar activity to chemo pharmaceutics, such as metronidazole. Their quantity was less than 10% of the extracted AMPs, and their mass ranged from 4.17–4.25 kDa [106]. The masses of this peptides were within the range of the AMP masses with anti-*H. pylori* activity extracted from other organisms (i.e., amphibians and fish with 1.99–4.40 kDa) [107].

Antimicrobial properties were also associated with larval fat. Some fatty acids, e.g., lauric acid, have antimicrobial properties by disrupting the surface of the bacteria—the cell wall and membrane [108]. This acid is a major component in the fat profile of *H. illucens* larvae.

In the case of oil extraction from *H. illucens* for antimicrobial purposes, [109] suggested that the traditional mechanical pressing of dried larvae is the most efficient method to ensure no loss of antimicrobial activity. The extracted oil did not cause changes in the growth of Gram-negative bacteria but inhibited Gram-positive bacteria, such as *B. subtilis* and *S. aureus*.

The high antimicrobial resistance of *H. illucens* may be influenced by the presence of live fungi in their digestive tract. Correa et al. [110] isolated 25 different fungal species from the gut of *H. illucens*, fed with guano from chickens. The highest antimicrobial activity (tested for MRSA ATCC 43,300 and *Salmonella typhimurium* ATCC 13311) was recorded for *Chrysosporium multifidum*. Seven antimicrobial compounds were isolated: six α-pyrone derivatives and one diketopiperazine. The α-pyrone derivative no. 4, limited the growth of MRSA at MIC 62.5 μg·mL^−1^ [110].

Other compounds with antibacterial and antifungal activities isolated from *H. illucens* are ommochromes, pigments from the flies’ eyes. Dontsov et al. [111] investigated those compounds suspended in 0.1 M potassium-phosphate buffer solution. Tests were performed on *B. subtilis* ATCC 6633, *Candida albicans* ATCC 2091 and *A. niger* INA 00760, noting the zones of growth inhibition of these microorganisms [111].

Much different approach has been presented by Saadoun et al. [112]. They proposed lactic acid fermentation of the pupae, prepupae and dead flies of *H. illucens* using *Lacticaseibacillus rhamnosus* 1473 and *Lactiplantibacillus plantarum* 285 [112]. In the case of *L. monocytogenes* and *Salmonella*, the best antimicrobial results were observed for fermented puparia. A similar decrease in the growth of these bacteria, as well as *E. coli*, to near 1 log CFU·g^−1^ was also observed for fermented flies [112].

In recent years, research has focused on deciphering the *H. illucens* transcriptome, which may provide an interesting source of information, relevant for increasing AMP production [113]. In silico studies showed that the transcriptome in both the larvae and flies contained 68 genes encoding AMPs, 57 of which encoded putative active peptides. Peptide sequence analyses showed that some of these exhibited antimicrobial, antiviral, antitumor or antifungal activity. Among the identified peptides, defensins was the most abundant (44%), followed by cecropins (18%), lysozyme (18%), attacin (7%) and the remainder (13%). To confirm the results of the in silico studies, four putative AMPs, which should possess the highest antimicrobial activity, were selected, and synthesized to conduct laboratory trials. A reduction of *E. coli* cell growth was found at a peptide concentration of 3 μM, with rapid bacterial death observed at 12 μM [113].

### 2.5. Biodiesel Production

*H. illucens* are capable of eating a wide variety of waste biomass thus offering the possibility of their revalorization into fat. The crude fat content of *H. illucens* larvae varies depending on the type of diet and can be up to 40% DM [114,115]. It can become a substrate for biodiesel production. The developmental stages of *H. illucens*, which have the highest fat levels, are late larvae and prepupae. Interestingly, for the next stage—pupae—an approximate 2.8-fold decrease in fat content was recorded [116].

FAME are components of plant-based biodiesel and can also be synthesized by transesterification from triacylglycerols extracted from *H. illucens* larval biomass. Insect biodiesel is qualitatively comparable to that of plants, produced from rapeseed oil [117]. Biodiesel based on *H. illucens* larvae consists of a large number of saturated fatty acids, while the unsaturated fatty acid content is lower, having a positive effect on the quality of biodiesel, i.e., increasing the viscosity and decreasing the autoxidation processes [118].

The larvae of *H. illucens* reared on various substrates, such as wheat grain [119], fruit waste, sewage sludge, palm decanter [120] or exo-microbial fermented coconut endosperm waste [115] had different FAME contents, but in each of these cases, lauric acid (C12:0) was their main component (Table 3). Spranghers et al. [18] reported that the overall fatty acid composition of *H. illucens* prepupae, which were fed four different diets (restaurant waste, vegetable waste, biogas digestate and chicken feed) also remains unchanged, with the lauric acid as the main component, even though the feed substrates contained low amounts of this acid [18].

Although [121] reported methyl oleate was the main fat component from larvae reared on solid restaurant waste, in most studies it is lauric acid. Ewald et al. [122] reared *H. illucens* larvae on 11 different diets, obtained lauric acid content 2.08 times higher than oleic acid. They found that the manipulation of fatty acid composition by changing the diet was irrelevant [122]. It has also been noted that with the increase in the weight of the larvae, there is an increasing tendency for the accumulation of saturated fatty acids [122].

**Table 3:** The content of the main component FAME depending on the feed on which *H. illucens* was reared.

Crude Fat Content (%)	Main Acid Residue of FAME	Content of Main Component of FAME (%)	Diet	Development Stage of *H. illucens*	References
33.6 ^a^	lauric acid	57.4	chicken feed	prepupae	[18]
21.8 ^a^	lauric acid	43.7	biogas digestate	prepupae	
37.1 ^a^	lauric acid	60.9	vegetable waste	prepupae	
38.6 ^a^	lauric acid	57.6	restaurant waste	prepupae	
35.0–40.0	lauric acid	65.7	exo-microbial fermented coconut endosperm waste	larvae	[115]
23.3	lauric acid	35.6	dairy manure	larvae	[117]
-	lauric acid	38.4	wheat grain	larvae	[119]
-	lauric acid	58.3	sewage sludge	larvae	[120]
-	lauric acid	76.1	fruit waste	larvae	
-	lauric acid	48.1	palm decanters	larvae	
39.2	oleinic acid	27.1	solid residual fraction of restaurant waste	larvae	[121]
57.8	lauric acid	51.8	bread	larvae	[122]
46.7	lauric acid	28.6	fish	larvae	
40.7	lauric acid	39.9	food waste	larvae	
33.1	lauric acid	52.1	fresh mussels	larvae	
11.2	lauric acid	13.4	ensiled mussels	larvae	
29.7	lauric acid	32.3	rotten mussels	larvae	
20.4	lauric acid	47.4	bread and mussels 10%	larvae	
19.6	lauric acid	47.6	bread and mussels 20%	larvae	
17.9	lauric acid	43.6	bread and mussels 30%	larvae	
17.9	lauric acid	42.0	bread and mussels 40%	larvae	
16.1	lauric acid	35.3	bread and mussels 50%	larvae	
35.7–39.6	lauric acid	27.8	restaurant solid waste and exo-microbial fermented rice straw	larvae	[123]
-	lauric acid	44.9	food wastes from cafeteria	prepupae	[124]
31.2	lauric acid	87.46	chicken manure mixed with rapeseed straw	larvae	[125]

^a^ calculated based on data provided in the publication.

Research indicates that the method of killing the larvae is an important step when obtaining fat for the purposes of biodiesel production. It affects the quality of fat in terms of total moisture, lipid content, oxidation state, pH and the presence of microbial populations [126]. Larouche et al. [126] found that the most lipid-efficient method is asphyxiation by vacuum packing and holding in an atmosphere of 100% CO_2_ or N_2_.

The purpose of lipid extraction is also important. Killing the prepupae by freezing has been shown to activate the action of lipases, thus contributing to the release of fatty acids. Blanching of the prepupae resulted in a more stable lipid fraction [127]. However, ethical considerations should be kept in mind and the method used should not increase the suffering of the insects unnecessarily.

The content and quality of lipids is extremely important for efficient biodiesel production. The number of lipids extracted from *H. illucens* larvae can be increased by the addition of microorganisms and larval feed fermentation. As demonstrated by [115], inoculation of coconut endosperm waste with 0.5% DM of the commercial septic system purification powder, RID-X (Reckitt Benckiser, Parsippany, NJ, USA) containing natural bacteria and enzymes as a fermentation initiator, increased the total amount of lipids extracted from the larvae by 35–40% [115]. This was probably associated with a higher availability of nutrients, which also saved the metabolic energy of insects when digesting the feed. Another example has been reported by [125], who showed that the *H. illucens* larvae had the highest extractable lipid content (approximately 31.8% DM) when the insects were fed with chicken manure mixed with solid digestate from the methane fermentation of rapeseed straw at a ratio of 1:3.

Enzymatic treatment of the feed can be another way to improve quality of the fat. Lim et al. [128] reared *H. illucens* on pretreated palm decanter cake waste with cellulase. The highest SFA content in the fat was 82.14% under treatment with 1% cellulase for 48 h. A high SFA content characterizes good quality biodiesels, affecting oxidative stability and kinematic viscosity.

The solvent used in extraction also had a huge impact of the efficiency of the process. Ravi et al. [129] conducted the screening of different solvents used in isolation of the lipid components. Screening included n-hexane, alcohol solvents (ethanol, iso-propanol), esters (DMC, methyl acetate, ethyl acetate, ethyl lactate), ethers (2-MeO, CPME) and terpenes (α-pinene, D-limonene, p-cymene). The most efficient solvent was 2-methyloxolate (2-MeO) with a total oil yield of 35.83%. For n-hexane, a standard solvent in lipids extraction, the oil yield was 32.51% [129].

To increase the lipid extraction efficiency (up to 75.9%), it was suggested by [130] that the biomass of *H. illucens* larvae should be pre-disrupted by high-speed homogenization, followed by biphasic extraction by means of isopropanol and petroleum ether at a volume ratio of 3:5. Among the solvents tested, such as n-hexane, petroleum ether, ethanol and isopropanol and their various mixtures, the binary solvents were found to be almost twice as efficient in lipid extraction as the monophasic solvents. For example, the binary solvent consisted of isopropanol and petroleum ether in a 1:1 ratio resulted in a lipid extraction efficiency of approximately 74.2%. The use of these solvents separately resulted in efficiencies of 34.7 and 51.4% for isopropanol and petroleum ether, respectively [130].

Transesterification is a key step in the production of biodiesel, commonly carried out with a catalyst. For instance, [117] conducted a two-step lipid conversion process to produce biodiesel from *H. illucens* larvae fat, consisting of H_2_SO_4_-catalysed pretreatment and NaOH-catalyzed transesterification. The final yield was 15.8 g biodiesel from 70.8 g dry *H. illucens* larvae, which was 22.32% [117]. Optimizing the conditions affecting the transesterification process in overall biodiesel production can be found in a review by [131].

Transesterification can also be conducted with the use of free or immobilized lipases. The use of free lipases is more economical, however, it may give rise to various by-products [132]. He et al. [132] proposed a method to produce biodiesel from *H. illucens*, using a Lipase Eversa Transform 2.0 with Lipase SGM1. After optimizing the transesterification conditions, they obtained results showed that the best ratio of *H. illucens* larvae lipids to methanol was 1:3, and the reaction temperature should be 25 °C. They obtained a FAME yield of 98.45%, an acid value of the biodiesel of 0.10 mg KOH·g^−1^ and a viscosity level of 4.57 mm^2^·s^−1^ [132].

A more recent study produces biodiesel through non-catalytic transesterification. The process was carried out in a bulkhead reactor filled with silica, methanol, and *H. illucens* larvae lipids extract for the first run, and dried *H. illucens* larvae powder instead of lipids extract on the second run, named as the direct non-catalytic process of the reactor was heated to 390 °C for 1 min and then cooled. The biodiesel yield from the *H. illucens* larvae lipids extract and from the dried and powdered *H. illucens* larvae was 34.0% and 34.7%, respectively, similar to the common base-catalyzed reaction (33.9% yield) [133].

Regarding the practical use of *H. illucens* for the purpose of running diesel engines, two strategies can be utilized, the use of crude insect oil or the use of its methyl esters. Kamarulzaman et al. [134] conducted practical tests of using crude oil from *H. illucens* larvae and their admixtures with petroleum-derived diesel fuel in a direct injection engine and investigated the combustion, performance, and emissions characteristics. It was found that the kinematic viscosity of *H. illucens* larvae oil was 8.4 times higher than standard diesel fuel. Its density was also higher (1.1-times) but its volatility was lower. The flash point of the oil was very high (>399 °C vs. 60 °C for standard diesel fuel). Those characteristics are very important as they influenced the atomization and fuel-air mixing rate. Due to its non-optimal properties, *H. illucens* larvae oil exhibited a poorer performance as diesel fuel (e.g., the peak cylinder pressure and heat release rate was lower by 3.28% and 13.38%, respectively). As the amount of *H. illucens* larvae oil increased, the amount of CO, CO_2_ and hydrocarbons produced also increased (by 17.5%, 13.6% and 474.3%, respectively). However, a reduction of NO_x_ and O_2_ concentration was observed (by 19.6% and 1.8%, respectively) [134].

Rehman et al. [135] studied two doses (10 and 20%) of *H. illucens* larvae biodiesel as an additive to standard diesel fuel. Biodiesel from *H. illucens* larvae has more similar characteristics to diesel fuel than crude *H. illucens* larvae oil: its kinematic viscosity was only 1.3-times higher than petroleum diesel fuel, and its density was only 1.05-times higher. The researchers found that the heat release rate increased with higher *H. illucens* larvae biodiesel addition. NO_x_ emissions increased with the addition of *H. illucens* larvae biodiesel, as well as general fuel consumption in comparison to standard fuel. When *H. illucens* biodiesel was added in amount of 20%, smoke intensity and fuel consumption were reduced in comparison to a 10% addition [135]. Current environmental conditions and the associated climate crisis demands for potential, alternative, and environmentally safer energy and fuel sources, thus insect oil is definitely worth future investigation.

### 2.6. Biogas Production

The development of the industry dealing with the breeding of insects for fodder and nutritional purposes, which has been studied in recent years, will be conducive to increasing the amount of waste generated during the breeding process [136]. Direct types of post-production waste, consisting of feed leftovers and insect excrements can easily be used as plant fertilizer [137]. Recently, the use of the generation of energy in the form of biogas through anaerobic digestion has also been explored [136,138].

Lalander et al. [138] conducted a study on producing biomethane from *H. illucens* larvae post-breeding residues in relation to food waste and human feces. Mesophilic fermentation lasted 16–22 days at 37 °C. The ratio of substrate to inoculum was 1:3 based on VS, i.e., the content of organic mass. The results of biomethane potential (BMP) are expressed as mL CH_4_ g^−1^ VS in Table 4. There was a decrease in BMP on the substrates after *H. illucens* larvae culture. Larval treatment for the fecal variant reduced the result by 1.89 times and 1.25 times for food waste. These results may have been due to the low vs. content and C:N ratios in the larvae-treated materials, which was the effect of larvae feeding and assimilating the nutrients from the substrate [138].

Bulak et al. [136] performed gasification of the post-breeding residue of *H. illucens* larvae, reared on carrot-beet marc (3:1 *v*:*v*) with an inoculum to substrate ratio of 2:1 (VS-based) at 37 °C for 21 days. The BMP values, as well as the CH_4_ concentration they obtained, were similar to the values obtained by [138] in relation to *H. illucens* larvae treated human feces (Table 4). These results approximated the BMPs of fruit and vegetable waste (153–342 mL CH_4_ g^−1^ VS) and poultry manure (107–342 mL CH_4_ g^−1^ VS) [139]. Compared to the above results obtained by [138,140], who also reared *H. illucens* larvae on food waste (fruit and vegetable peelings and seeds and food leftovers), obtained BMPs 1.56 times higher (Table 4). The post-breeding waste obtained by [140] had a lower TS content with a higher VS. (% of TS).

Another possibility of biogas generation is the fermentation of whole insects or their parts, e.g., a cuticule which is left after lipid extraction from the larvae [140]. The experimental variants investigated by [140] involved the use of: unchopped larvae reared on organic waste; larvae reared on chicken feed (CF) and food waste (FW) chopped, ground and sieved to achieve a particle size distribution between 90 and 250 µm; extracted *H. illucens* larvae lipids, the naturally dead flies, dried and ground larval cuticule that remained after manual extrusion of inactivated larvae, larval feeding residues. The highest results of more than 600 mL CH_4_ g^−1^ VS. were obtained for larvae fed both chicken feed and food waste, which were cut and grounded (Table 4). Interestingly, the average BMP from *H. illucens* larvae was higher than that obtained from more traditional methane fermentation substrates such as energy crops. For example, the BMP from ryegrass was 140–360 mL CH_4_ g^−1^ VS and from switchgrass, 122–246 mL CH_4_ g^−1^ VS [139]. This observation was also supported by [141], who studied BMPs from larvae fed on plant waste (Table 4).

**Table 4:** Biomethane yield obtained from the fermentation of different raw substrates obtained from *H. illucens* or its wastes.

	Feedstock	Cumulative Biomethane Potential (mL CH_4_ g^−1^ VS)	CH_4_ Concentration (% vol.)	Reference
Whole insect or its parts	* H. illucens * larvae-food waste	675 ± 118	n.d	[140]
	* H. illucens * larvae-chicken feed	661 ± 29	n.d.	
	dead flies	570 ± 51	n.d.	
	lipid extracted *H. illucens* larvae-food waste	363 ± 32	n.d.	
	larval cuticule	343 ± 7	n.d.	
	lipid extracted *H. illucens* larvae-chicken feed	306 ± 23	n.d.	
	whole *H. illucens* larvae	108 ± 65	n.d.	
	whole *H. illucens* larvae	455.87 ± n.d.	64.27	[141]
* H. illucens * frass	* H. illucens * larvae post-breeding waste	207.9 ± 21.5	53.2 ± 3.2	[136]
	* H. illucens * larvae-treated human faeces	178.9 ± 7.1	55.2 ± 0.7	[138]
	* H. illucens * larvae-treated food waste	322.6 ± 6.4	61.4 ± 0.4	
	* H. illucens * residues	502 ± 9	n.d.	[140]

n.d.—no data.

### 2.7. Entomoremediation

The idea of entomoremediation first appeared in the literature in 2013, with the theoretical publication of [142], who considered the application of collembolans, ants, beetles (e.g., dung beetles) and termites for the cleaning of degraded soil. The prefix (gr.) “entomon” means an insect, and (lat.) “remediation” is the process of cleaning or restoring the lost properties of the environment. Entomoremediation was defined as the use of specialized insects and their associated microorganisms to utilize, extract, sequester and/or detoxify pollutants from the soil, sediments, and organic biomass [143].

Entomoremediation is of course not exclusively connected with the *H. illucens* larvae but, as this research field is very new, most of the research directly regarding this topic was carried out on *H. illucens*. More time and research are still needed in order for this word to be accepted in the general consciousness of researchers, therefore many publications that deal with this topic do not use the term “entomoremediation” but rather more general words relating to the bioremediation *sensu lato*.

In the case of the entomoremediation of inorganic pollutants (like toxic heavy metals), which can be referred to more specifically as entomoextraction, the use of a specific insect for this purpose must be associated with its ability to bioaccumulate these pollutants, in order to ensure its transfer from the environment to the insect body. In the case of *H. illucens*, older studies have focused on the phenomenon of bioaccumulation of heavy metals in different developmental stages, in the context of insect physiology and toxic effects [144,145,146,147]. Other studies, conducted in the context of the nutritional properties of *H. illucens*, have concentrated on the content of elements, primarily in two developmental stages, which could be used for the production of insect fat and proteins for feed purposes: larvae and (pre-)pupae [18,148,149]. Regardless of the context, all of these studies provide an insight into the bioaccumulation potential of *H. illucens*.

These research studies showed that metals, in particular, Cd, Zn and Pb were bioaccumulated from optimal (“model”) feed, which was chicken feed or wheat bran spiked exogenously with metals [144], even when the concentrations of those elements were lower than permissible limits [145]. Gao et al. [146] found that contrary to Cd, Cr did not accumulate into any of developmental stages of *H. illucens*. The concentrations of metals, in general, decreased with the successive development stages of the insect, which was consistent both with earlier and later studies [143,144]. Spranghers et al. [18] investigated the mineral composition of *H. illucens* prepupae on different substrates, which were chicken feed, digestate from the methane fermentation of vegetable waste, vegetable waste and restaurant waste. These studies showed a bioaccumulation of Ca, Fe, Mg, Mn, P, and Zn, however, overall concentrations in the *H. illucens* were rather low.

Currently, in laboratory experiments, a more precise measurement of bioaccumulation (BAI—bioaccumulation index, [150]) has been proposed. It should also be remembered that bioaccumulation is dependent upon the type of substrate and the initial concentration of the elements [143]. Heavy metals were mainly bioaccumulated in the larvae and pupae, as well as in puparia, which are empty pupae shells. Empty puparia constitute very interesting materials in the context of entomoremediation, as they can contain a relatively high concentration of heavy metals, are dry and light, and can be easily obtained during *H. illucens* growth [143]. As pointed out by [146] if the concentrations of a given heavy metal is sufficiently high, puparia facilitate attempts to recover these metals.

Large quantities of heavy metal-polluted plant biomass are generated during the process of phytoextraction, i.e., the use of plants (specific hyperaccumulator plant species or fast growing species, like corn) to extract metallic pollution from the soil and sequester it in its shoots, which can then be harvested [151]. One of the major drawbacks of this method is that such polluted biomass can pose a threat and must be stored in a hazardous waste landfill (hazardous effluents) or neutralized with the use of technologies that require equipment and energy expenditure [152]. The use of saprophagous insects with bioaccumulation abilities provides a new approach for the utilization of this type of biowaste. Bulak et al. [143] proposed the use of *H. illucens* for the entomoremediation of corn leaves, polluted with Cd and Zn. During the 36 days of the experiment, DM utilization of contaminated corn leaves was 44.4% for Cd and 50.0% for the Zn variant, which was better than the standard composting of contaminated plant biomass in terms of both biomass utilization and time. A high Cd concentration has been noted in the larvae and in the puparia, while the Zn in the larvae and the imagoes of *H. illucens* indicated differences between essential and toxic element physiology [143].

When *H. illucens* larvae were fed solely on solid aquaculture, their waste contained heavy metals and trace elements at levels unacceptable for use as a feed material; the larvae showed bioaccumulation of Cd (BAF 2.5–2.7), Hg (BAF 1.6–1.9), Mn (BAF 1.3), as well as the first reported bioaccumulation of Ag (BAF 0.9–1.3) [153].

A study of the bioconversion of heavy metal-rich biosolids by *H. illucens* larvae did not report a high bioaccumulation of elements in their bodies [154]. In fact, the survival ratio was around 83%, but this was related to the low number of nutrients in the substrate. A BAF value in *H. illucens* larvae-fed biosolids that was greater than 1, was recorded for Ca (approx. 5.3), Mn (approx. 1.7), Cd (approx. 1.8) and K (approx. 3.8). A reduction in waste mass can be regarded as a positive effect [154].

Proc et al. [149] investigated the potential of *H. illucens* to bioaccumulate different elements from the optimal feed (not artificial contaminated). It was found that Ca, Cd, Ga, Mn, P and S were only bioaccumulated in certain developmental stages of *H. illucens*. Conversely, elements like Ba, Bi, Cu, Fe, Hg, Mg, Mo, Se, and Zn bioaccumulated in all stages and puparia. The study also revealed that Al, As, Co, K, Pb, and Si did not bioaccumulate at all [149].

*H. illucens* is known as an insect which is rather resistant to toxic heavy metals in its feed. The addition of Cu and Zn did not result in differences in larval length and the average fresh weight (FW) of the individual larvae remained within a range reported in the literature (34–315 mg FW) [143,145,147]. However, Cd can cause an increase of the DM of individual larvae as shown by [143,144,147]. Some negative effects were evident in relation to the growth performance of *H. illucens*—the addition of Cd and Pb caused a slight increase in the development time of *H. illucens* larvae as reported by [144,145]. Bulak et al. [143] demonstrated a four-time increase in the mortality of the larvae when fed on Cd or Zn polluted corn leaves.

An important aspect regarding the bioaccumulation and bioavailability of heavy metals is the larval density factor. Jiang et al. [155] optimized the inoculation density of larvae fed on swine manure, using 0.08%, 0.24% and 0.40% larvae addition in FW. It was noted that the larvae from the 0.40% variant accumulated the highest amount of metals. A particularly high result was obtained for Cd, which had a bioaccumulation factor of 23.5, an increase of 6.6 times compared with the 0.08% variant [155].

Recently, much attention has been paid to the ability of *H. illucens* to degrade organic pollutants, which can be termed entomodegradation. Regarding *H. illucens*, such studies were conducted for contaminants like mycotoxins [156,157], hydrocarbons [158], insecticides [159] and antibiotics [160].

No mycotoxins were detected in *H. illucens* larvae reared on feed that was corn contaminated with deoxynivalenol (DON), fumonisin 1 and 2 (FB1 and FB2) and zearalenone (ZEN)[156]. This suggests the possibility of entomodegradation of plant biomass contaminated within these dangerous toxins [156]. Another study revealed that *H. illucens* larvae are capable of feeding on aflatoxin B1 contaminated biomass. Meijer et al. [157] proved that cytochrome P450 and cytoplasmic reductase were involved in the detoxification of aflatoxin B1 to aflatoxicol and aflatoxin P1, with an efficiency rate of around 60%.

*H. illucens* larvae can also survive on feed with added polycyclic aromatic hydrocarbons (PAHs) such as naphthalene, flourene, phenanthrene, and pyrene [158]. Moreover, it was reported that changes in the concentrations of these PAHs showed no significant differences in *H. illucens* larvae survival, harvest, conversion and eclosion rates. The degree of PAH removal by *H. illucens* larvae depended on the concentration of PAH in the substrate (1, 10, 100 mg·kg^−1^). During the experiment, which lasts 18–21 days, the removal rate for naphthalene was 34.1–84.2%, for fluorene 63.9–83.1%, for phananthrene 48.0–62.9% and for pyrene 52.2–74.6% [158].

A study was conducted to check the survival parameters of *H. illucens* insects, as well as their bioaccumulation ability on food containing insecticides. Meijer et al. [159] used insecticides such as chlorpyrifos, propoxur, cypermethrin, imidacloprid, spinosad, tebufenozide, cypermethrin with and without piperone butoxide (PBO) and administered them in the feed as initial doses, corresponding to the European Union maximum residue level (MRL), to evaluate the mortality and growth of *H. illucens* larvae [159]. The insecticide concentrations were then adjusted for further testing, using higher or lower doses, based on the preliminary results of whether the product was accumulated by the larvae. The final dosages were: 0.5 mg·kg^−1^ chlorpyrifos, 0.5 mg·kg^−1^ propoxur, 1 mg·kg^−1^ imidacloprid, 0.2 mg·kg^−1^ spinosad, 0.5 mg·kg^−1^ tebufenozide, 0.1 mg·kg^−1^ cypermethrin, 2 mg·kg^−1^ PBO, and 0.1 mg·kg^−1^ + 2 mg·kg^−1^ cypermethrin + PBO. The survival rate of the *H. illucens* larvae compared to the control was not significantly different, except for the trials with spinosad, cypermethrin, and cypermethrin mixed with PBO. It was observed that the larvae treated with an imidacloprid diet increased their biomass relative to the control. The insecticides concentrations in *H. illucens* larvae were at such a low level, that bioaccumulation was considered not to occur [159]. The lack of insecticide accumulation on the part of the larvae is beneficial if it is to be applied as feed, as it would be free from chemical contaminants that might be present in the diet substrate given to *H. illucens* during rearing.

Antibiotics are another xenochemical studied. Liu et al. [160] used three doses of oxytetracycline: 100, 1000, and 2000 mg·kg^−1^ on a DM basis, and after 8 days of the experiment, its degradation efficiency after *H. illucens* larvae treatment was 82.7%, 77.6% and 19.3%, respectively. As in the previously described work, there was no significant effect on larval survival, which indicates that *H. illucens* larvae are very resistant [160].

Another example of an antibiotic biodegradable by *H. illucens* larvae is lincomycin [161]. Studies were carried out to check the degree of reduction of lincomycin fermentation residues (LFR) + wheat bran (1:1), as well as the biodegradation of lincomycin by *H. illucens* larvae (wheat bran + lincomycin hydrochloride solution (1000, 1500 and 2000 mg·kg^−1^ DM)). The total mass of LFR decreased by 67% DW after 12 days, while the degree of lincomycin degradation was recorded at approximately 84.9%. The presence of this antibiotic in the body of *H. illucens* larvae was also not detected [161].

### 2.8. Insect Frass

To ensure high soil productivity a continuous supply of mainly N, P and K have to be maintained in the form of fertilizers. In the EU, natural fertilizers in the form of cattle manure are more and more difficult to access, due to the reduction of the number of these animals and high legal requirements related to the maintenance of herds, which particularly affects individual farmers. Increasing adverse climatic changes are likely to force a reduction or a shift away from beef or swine consumption, due to the emissions of greenhouse gases during breeding, which will further reduce herds [162]. It is at this stage that insects, such as *H. illucens*, come to the fore, as they consume their feed, which can often be waste biomass, assimilate essential elements and return it to the environment in the form of insect excrements, which may provide a new type of natural fertilizer [163].

Insect frass, as defined in European Commission Regulation 2021/1925 [164], is “a mixture of excrements derived from farmed insects, the feeding substrate, parts of farmed insects, dead eggs and with a content of dead farmed insects of not more than 5% in volume and not more than 3% in weight”.

According to the Regulation, insect frass can be introduced to the market after heating it up to 70 °C for one hour, which correlates with the requirements also in place for processed manure. Such products can already be found on the market, although research into how insect frass works on plants and the environment is still ongoing. An example of a commercial fertilizer already being used is HexaFrass™ (HexaFly, County Meath, Ireland), which is produced after rearing *H. illucens* larvae mainly on brewery waste [165]. The averaged content of the main nutritional elements for plants in different *H. illucens* larvae frasses was around 3.39% DM for N, 2.85% DM for P_2_O_5_ and 3.47% DM for K_2_O [166].

Recent studies considered the effect of the aforementioned heat treatment (70 °C for 60 min) on the microbial abundance of *H. illucens* frass [167]. There was a negligible decrease in the total abundance of viable microorganisms and bacterial endospores, however, a decrease in *Enterobacteriaceae* below the detection level was registered. In the case of frass, inoculated with foodborne bacteria like *Salmonella* spp. and *Clostridium perfringens* (5.0 log CFU·g^−1^), heating caused its complete reduction [167]. The study proved the validity of the frass procedure of sanitization, as the pathogenic bacteria for the animals were killed.

A very new and interesting research area investigates how frass influences phytopatogenic microorganisms. Gebremikael et al. [168] showed that the addition of three different *H. illucens* frasses (without thermal treatment) reduces the number of pathogenic *Rhizoctonia solani* in the soil. They highlighted that the addition of frass from general food waste and from vegetable waste in tests on beans (*Phaseolus vulgaris*) infected with this pathogen, resulted in a decrease in disease rates by approximately 50%. The researchers speculate that the reduction of *R. solani* may have been caused by chitinase activity [168].

In contrast, [93] found the presence of bacteria from the family *Xanthomonadaceae*, in which there are some plant pathogens, when investigating *H. illucens* frass from organic household waste. Consequently, there is a potential threat to plants. More studies are needed to check whether the compliance with the recommendations contained in the Commission Regulation (EU) 2021/1925 [164] can eliminate this risk of phytopathogens, spread by the use of *H. illucens* frass.

Another recent study also considered the antifungal properties of frass extracts from *H. illucens* culture on two types of substrates: fruit/vegetable/bakery/brewery waste (FVBB) and a standard Gainesville (GV) diet. The effect of frass extract filtration (nylon filter, 0.45 µm) was also investigated. The unfiltered GV extract showed high inhibition for all tested mycelium—*Alternaria solani*, *Botrytis cinerea*, *Fusarium oxysporum, Pythium capsici*, *R. solani, Sclerotinia sclerotiorum*. In contrast, the unfiltered FVBB extract inhibited solely the growth of *B. cinerea*, *S. sclerotiorum* and, to a lesser extent, *A. solani*. Filtered extracts from both frasses did not show any changes in mycelial growth [169].

It is important to note that the composition and quality of frass is shaped by the food the larvae were fed [166]. Klammsteiner et al. [170] showed that the frass C:N ratio was higher when grown on fruits and vegetables (26.6) than when grown on grass (18.2) or chicken feed (18.5). When comparing the NH_4_NO_3_ frass with the *H. illucens* with nitrogen-equivalent amounts, a comparable increase in the growth of perennial ryegrass was noted (*Lolium perenne*). This frass also had a high total microbial load (up to 10^9^ CFU g^−1^) [170]. Interestingly, except for the Gram-negative bacteria, the frass from the chicken feed was also abundant with *E. coli* and coliforms, while in other types of frass, only Coliforms bacteria and Gram-negative bacteria were present. After the addition of those frasses to the soil the CFUs decreased and only Gram-negative and Coliforms bacteria were detected, with a much lower load of 10^3^–10^5^ CFU g^−1^, indicating that frass fertilizers did not affect the soil in a negative way in terms of hygiene [170].

Fischer and Romano [171] tested the frass obtained from *H. illucens* larvae reared on four substrates. These were fruit mixture, vegetable mixture, starch mixture and a as a last variant, a mixture of all three substrates in equal parts. The experiments lasted two weeks in a darkened room (34 °C, 70–80% moisture content). The highest NPK result was obtained for vegetable frass, which was 4.76:0.83:5.34. This was 1.58, 2.31 and 2.36 times higher than the ratio in the substrate before the experiment [171].

The *H. illucens* larvae treatment of different types of manure was found to improve the quality and stabilize the resulting product by improving the humification processes [11]. A nine-day experiment on chicken manure (CM), pig manure (PM), and cow manure (COM) resulted in a decrease in the initial protein content and an increase in the humic matter. The results showed that the degree of humification in the product after *H. illucens* larvae treatment were 42.45% for PM, 57.07% for CM and 63.74% for COM, and were higher than the control (manures without the addition of larvae) by 1.02, 1.34, and 1.27 times, respectively [11].

*H. illucens* frass can also be produced from the organic municipal waste from domestic, market and restaurant waste, as investigated by [172]. They noted an increase in the N, P, and K content in the frass of 41.2%, 32.4%, and 77.1%, respectively, as compared to the initial waste. The authors also investigated the bioaccumulation of various heavy metals. They found very high removal efficiencies for As (92–98%), Cd (99.4–99.9%) and Pb (80–90%) due to the bioaccumulation in the insect, however the initial concentration of these elements was below 1 mg·kg^−1^. With the higher initial content of Fe in the range of 3.4–5.6 mg·kg^−1^, the removal efficiency was lower (31.1–69.1%) than for toxic heavy metals. It is important to note that *H. illucens* larvae have the ability to bioaccumulate (see Section 2.7.), therefore, care should be taken when feeding the larvae with food that may contain increased levels of toxic elements, which may end up in the frass.

Research has also been carried out on the quality and effectiveness of various substrate additives or treatments that would positively affect the composition and properties of frass. One of the examples can be the inoculation of a substrate with microbial preparation. It has been found that for more efficient substrate conversion and to obtain nutrient-rich frass, the addition of *B. subtilis*, isolated from the guts of the *H. illucens* larvae may be used [173]. In the experiment, the latter used fresh chicken manure as the food substrate for the larvae and inoculated it with *B. subtilis* inoculum (10^9^ CFU·mL^−1^). The process of *H. illucens* larvae conversion lasted 13 days, followed by 11 days of aerobic fermentation. The final product was stable and characterized by a higher maturity, as a result of the enzymatic activity change during aerobic fermentation stage. The activities of catalase and polyphenoloxidase in *H. illucens* frass with bacterial inoculum continued to increase until the middle of the fermentation period, and their values were higher than in the control (*H. illucens* frass without inoculation). The peak value of urease activities did not differ significantly between the samples, while invertase activity was higher in the control. After 12 days of fermentation, each enzyme activity was undetectable or maintained at a very low level of activity, which indicated that the microbes were in a steady state. The high germination rate of Chinese cabbage (*Brassica rapa* L. subsp. *pekinensis*) and rapeseed (*Brassica napus* subsp. *napus*) also proved the maturity of the frass, after the aerobic fermentation stage in terms of no phytotoxicity [173].

The effect of the addition of gypsum (calcium sulphate) and biochar (made from rice husk at 350 °C) to a larval substrate consisting of brewery spent grain on the composting efficiency of *H. illucens* larvae and the quality of the resulting frass has been investigated by [174]. It was shown that the addition of gypsum at 10% DM resulted in the highest increase in N content of the frass, however, with the increasing dose of the additive, larval fresh mass and DM decreased. In the case of biochar, the addition of 20% DM gave the highest increase in N content of 21% in the frass and resulted in an increase in larval DM yield by 86%. In both cases, no significant differences were observed in the germination rate of cabbage seeds (*Brassica* sp.), which was around 90% [174]. Studies have also shown that the use of *H. illucens* larvae with the addition of biochar reduces the composting time from 8–24 weeks for conventional composting, to only 5 weeks [174].

In other research, Beesigamukama et al. [175] used sawdust from the blue gum tree (*Eucalyptus globulus*) to regulate the C:N ratio in the feed. Brewery spent grains with a C:N ratio of 15 contributed to an increased biomass conversion by *H. illucens* larvae, while at the same time increasing N (by 21%) and P (by 15%) retention in the compost. All frasses obtained in this research (with different C:N ratios) were not phytotoxic, as they had a seed germination percentage higher than 80%. Frasses with C:N 25 and 30 did not differ in comparison to the control in terms of this parameter (93.3% vs. 100% of germination in the control) [175].

The effect of *H. illucens* frass amendment on soils, e.g., nitrogen supply and its availability, was also investigated. Nitrogen release in soils fertilized with organic *H. illucens* fertilizer was slow, however, mineral nitrogen was increased, which may give a positive result of application [176]. When the *H. illucens* frass from brewery spent grains was used in a dose of 1.4 t ha^−1^, which equals 30 kg N ha^−1^ for corn (*Zea mays* variety H513) fertilization, this was sufficient for adequate N supplementation. The results were not statistically different in comparison to higher dosages of frass. Moreover, the yield of maize grains did not differ statistically in comparison to commercial organic fertilizer (SAFI Organics, Kenya) and was 4.96 t·ha^−1^ for *H. illucens* frass and 4.49 t·ha^−1^ for SAFI [176].

The addition of *H. illucens* frass from chicken manure to the soil, at a level of 4% (*w*:*w*) increased DM content in the above-ground parts of the rice (*Oryza sativa* variety E28) by 40.2%, increased the yield by 49.6% and the height and chlorophyll value by 11.9%, compared to the control with no fertilization. However, negative effects, such as a decrease in DM of the plants or a slowdown in their growth were noted with the *H. illucens* frass dose of 8% [177]. Such results could be due to, e.g., over fertilization of the plant or phytotoxicity of the fertilizer, however, the authors did not explain this result.

Gebremikael et al. [168] conducted research on the effects of the addition of three types of *H. illucens* frass from food waste, vegetable waste and chicken feed waste on soil. *H. illucens* frass from vegetables was used at a dose of 9.0 t FW·ha^−1^ and frass from chicken feed at a dose of 7.3 t FW·ha^−1^. These additions introduced 8% and 16% of total N into the soil, respectively, over a period of four months. In the case of *H. illucens* frass from food waste, an initial 91-day immobilization of N was noted. After that period, the presence of N increased to approximately 14%. However, these doses may be insufficient for most crops and may not bring the desired effects as a stand-alone fertilizer [168]. The researchers also noticed that the initial activity of dehydrogenase in soil with *H. illucens* food waste frass was 10 times higher than the control and decreased to 0 in 140 days. Enzymes in this group contribute to the oxidation of the organic fraction of the soil, which significantly affects soil fertilization and enhances soil quality.

Frass has also been tested as a direct cultivation substrate and as an additive for commercial peat in the cultivation of potted plants. Plants grown on a mixture of cultivation substrate and *H. illucens* larvae residue performed best with a ratio of commercial peat (Fondolinfa Universale, Linfa Spa, RE, Italy) 80% + *H. illucens* frass 20%; basil (*Ocimum basilicum* L., cv. ISI 602), tomato (*Solanum lycopersicum* L., cv. Roma V.F.) and lettuce (*Lactuca sativa* L., cv. Chiara) had higher total plant DM values by 1.19, 1.17 and 1.29 times, respectively, compared to the controls on commercial peat. A larger leaf surface and a greater number of leaves were also noted in the absence of any symptoms of abiotic stress [178].

A rather different approach to the use of frass was recently presented by [179], who investigated the fertilizing potential of a so-called compost tea, made from *H. illucens* frass. *H. illucens* larvae were bred on a mixture of coffee waste, dough, fish feeds, and fruit and vegetable waste. The frass was then mixed with water in a dose of 2.25 g·L^−1^ and used as an additive in an aquaponic culture of chili banana peppers and sweet potato. The additive increased the total sugar in the chili plant, while in the sweet potato, the Mn level was elevated in the leaves. The authors stated that *H. illucens* frass tea enhanced the nutritional quality of the plants overall [179].

### 2.9. H. illucens Larvae as a Food

Hunger is one of the world’s major problems. According to WHO [180] data from 2018, one in nine people are experiencing hunger. Additionally, the human population is also forecast to grow, which equates to an increasing demand for food. This will require finding an alternative, yet cheap source of protein. One of the solutions might be the consumption of insects, which is well known throughout the world. However, in developed countries, in particular, there is a stigma surrounding the consumption of insects, especially in their intact, raw forms. One of the candidate insects for human food, although less obvious, is *H. illucens*. This insect can quickly build larval biomass, rich in proteins and fats. Until now, this insect has been used for animal feed, however, research is being conducted to obtain safe food for humans.

Although *H. illucens* has not yet been approved for food in the European Union, studies have been carried out on its nutritional profile, which provide useful information on the possibility of use for this purpose. Mshayisa et al. [181] tested the nutritional properties and features of two types of *H. illucens* larvae flour: freeze-dried (BSFL-FD) and defatted (BSFL-DF), and two protein concentrate extraction methods: alkaline-acid (BSFL-PC1) (1 M NaOH, 0.5 h, 25 °C; 1 M HCl, overnight, 4 °C) and alkaline (BSFL-PC2) (1 M NaOH, 2 h, 40 °C). The highest protein content was obtained for BSFL-DF flour (50.12%), while in the case of protein concentrate, this was BSFL-PC1 (73.35%). The amino acid composition of these two variants were similar to cow’s milk and egg protein. The solubility of the protein at pH 2 was clearly higher for the protein concentrates and was approximately 85–97%. Protein concentrate had the best water-binding capacity, and showed the highest emulsion stability, while both flours had the best oil-binding capacity. The results proved that this protein extraction method yields a product with improved nutritional properties, with technical and functional characteristics [181].

Anankware et al. [182] tested a meal obtained from *H. illucens* and found that it was characterized by a crude fat content of 18.03% DM, which was 2.27 times lower, when compared to beef. Crude protein was 44.82% DM, which was 1.23 times less than beef. The contents of neutral detergent fiber and acid detergent fiber were 39.94% and 15.57% DM, respectively. SFA, mono-unsaturated fatty acids and PUFA content were 61.36%, 26.36% and 9.18%, respectively. Too high a level of SFA affects blood pressure and can lead to cardiovascular disease. Therefore, more research is needed regarding the safe use of *H. illucens* for human consumption [182]. By comparison, the SFA content of beef was determined to be around 36.7–46.3% of total FA [183]. Zozo et al. [184] reported 45.82% protein and 25.78% fat content in *H. illucens* flour. In addition, they found that the process of defatting the flour with the use of hexane:isopropanol mixture (3:2 *v*:*v*) increased its protein content by 1.2 times, while its fat content decreased by 5.3 times. The defatting process also affected the mineral composition of the flours; it caused an increase in the content of Fe, Mg, Mn, K, and Zn. There was also a 7.5-fold decrease in the Na content of skimmed flour, which can be beneficial for a low Na diet. The content of Mg, Mn, and Zn in the defatted flour was above the recommended daily intake, but this can still be very useful in terms of microelements in fortified foods. Both types of flours showed thermal stability properties [184]. These authors confirm the feasibility of using flour from *H. illucens* larvae in human food production, while suggesting the need for further studies, such as protein digestibility and defatting methods.

Bessa et al. [185] conducted a study focusing on safety in terms of microbiology, heavy metal content and allergens, when using *H. illucens* larvae as a direct-to-human food. They tested three different food substrates on which the larvae were reared: a broiler-based diet, brewers’ grain, and cereal grain. Two methods of killing larvae, namely, freezing and blanching were also tested. The lowest concentrations of *B. ceureus* and *E. coli* bacteria were recorded in blanched larvae reared on broiler-based feed; the blanched larvae also had the lowest element content. In the larvae killed by freezing, microbial concentrations were at the same level as in the feed, regardless of the variant. However, the larvae killed by blanching contained a higher content of allergens than when they were frozen. These allergens were tropomyosin and arginine kinase, which are also characteristic of crustaceans. Feeding the larvae did not affect the allergen content, only the heavy metal content [186].

Equally important in terms of sanitation and food safety was the absence of pathogenic viruses, which, to date, have not been found in any of the developmental stages of *H. illucens* [187].

### 2.10. H. illucens in Cosmetic, Cosmeuticals and Personal Care Products

*H. illucens* can be a source of components used in the production of cosmetics, as for example, proteins, chitin, chitosan, or fat. However, most of the articles on this topic are theoretical and are in the form of review suggestions, based on the characteristics of the properties of larvae [77,188,189].

In the case of proteins, it is generally known that certain amino acids, such as glycine and arginine, can be used in the formulation of cosmetics, i.e., due to their hydrating and antioxidant properties or their properties in collagen production. The content of these amino acids in *H. illucens* larvae vary with the type of diet on which they were reared, and their values can reach up to around 51 g·kg^−1^ of crude protein and 59.2 g·kg^−1^ of crude protein for arginine and glycine, respectively. In addition, the AMPs produced by *H. illucens*, due to their properties, can be used as an active ingredient in cosmetics for problematic skin [188].

Chitin, due to its antimicrobial, moisturizing and biocompatibility properties, can be used as an active compound in cosmetics. Chitin can be transformed into various forms, including, hydrogels or membranes [77] however, an interesting approach and one that has been popular in recent times is the use of nanotechnology and the production of nanofibers and nanofibrils, which can act more precisely as carriers of active agents [77].

Lipids from *H. illucens* are characterized by a fatty acid profile that is rich in lauric, myristic, palmitic and oleic acids. These FAs are also found in more conventional and cosmetically used fat sources such as coconut oil, palm oil and palm kernel oil [188,189]. It has been suggested that fatty acids from *H. illucens* can replace those from coconut oil or palm kernel oil and be used as an alternative source of these ingredients. Medium chain FAs express antimicrobial properties (see Section 2.4). The most important role here has lauric acid and its metabolite monolaurin (glycerol monolaurate), with confirmed antiviral, antibacterial and even antiprotozoal properties [188]. This compound is present naturally in fats and oils but in very low concentrations [190]. Recently Xu et al. [190] demonstrated its production via enzymatic glycerolysis from the oil of *H. illucens* larvae. It should be noticed that FAs along with monolaurin presents or produced form *H. illucens* can serve also in cosmetic formulations as emulsifiers, stabilizers, and conditioning agents [188,190].

Interestingly, Ushakova et al. [119] detected also low quantities of azelaic acids in *H. illucens* lipids. This is a well-known compound used in skin care cosmetics as it inhibits the reproduction of lipophilic microorganisms and therefore helps counteract infections [189].

Chou et al. [191] proved that nano-emulsion can be made from *H. illucens* oil. Nano-emulsion is a mixture of water, oil and emulsifiers obtained by physical homogenization. They produced nearly spherical nano-emulsion with long-lasting stability [191], which could be a base for cosmetics formulation.

In addition to studies on the FAs profile in *H. illucens*, Almeida et al. [192] conducted preliminary studies on the toxicity of FAs from *H. illucens* and its antioxidant activity, which could be also of interest from cosmetic production point of view. The results showed that the *H. illucens* lipid extract (with a concentration of 0.1 mg·mL^−1^) showed low antioxidant activity. The authors stated that it could be the effect of too much dilution of the extract and propose further research on this topic. Tests carried out on the organism *Artemia salina* (L.) showed a lack of toxicity of the lipid extracts [192].

Verheyen et al. [193] used extracted and refined fat from *H. illucens* to produce hand cream formulation and compared it with cream produced form mink and plant oils. The high content of lauric acid (>60%) made insect fat it less suitable for skin care product than both other tested oils. Insect fat contained also high amounts of free FAs and phospholipids, which must be removed prior to cosmetic production. Generally, from the physicochemical point of view they stated that *H. illucens* fat was suitable for leave-on cream preparation. Research focused on different refining methods of raw *H. illucens* fat from phospholipids and to improve the color and odor should be done, if one want to use it in real application in cosmetics [188,193]. Deeper studies on the presence of contaminants such as pesticide and solvents as well as more toxicological tests of real cosmetic formulations would be necessary in the future [193].

### 2.11. Bioplastics

In order to improve the Earth’s ecological situation and reduce the production of fossil fuels, new and more biodegradable bioplastics are constantly being researched. Recent studies showed that a type of bioplastic can be made from *H. illucens* proteins. One of the earliest works focusing on bioplastics production, derived from *H. illucens* proteins, was [194]. This study proposes the use of glycerol and citric acid as additives to *H. illucens* larvae protein isolate, which would act as a plasticizer and crosslinking agent, respectively. After optimization, the following ratio of bioplastic components was proposed: 13 g distilled water, 85% (*w*:*w*) glycerol and 0.5 g of *H. illucens* protein, without any use of citric acid. This dose of protein gave the best result in terms of extensibility value, but it limited the thickness of the material [194].

Nuvoli et al. [195] tested the whole protein fraction with *H. illucens* prepupae and their soluble fraction for biofilm formation. The use of soluble proteins resulted in biofilms that were more stretchable, strong, and transparent. The addition of citric acid as a crosslinking agent resulted in a decrease in tensile stress resistance by almost half; elongation at the break increased by around 1.16 times, however the citric acid reduced water absorption by the *H. illucens* biofilms. In contrast, the addition of CA improved the properties of biofilms from the whole fraction [195].

More recent work involved the practical testing of bioplastic film produced from *H. illucens* as a mulch [196]. The properties of the bioplastic film, e.g., degradability, weight and thickness of the film, water evaporation and soil microbial content (SMC) were tested and compared with commercial biodegradable film from corn starch Mater-Bi (Novamont S.p.A., Novara, Italy) and non-biodegradable polyethylene film [196]. In terms of water evaporation, the bioplastic from *H. illucens* achieved similar results to the commercial Mater-Bi film at around 0.19 g H_2_O day^−1^ cm^−2^. The film with *H. illucens* was the thickest (0.36 mm) and heaviest (0.84 g) of all the variants. Due to its completely organic composition, it exhibited the fastest degradation time and after just 10 days of mulching, the film area decreased by 1.23 times, and thickness and weight decreased by 1.03 and 1.25 times, respectively, while the control films remained intact. Analyses of SMC showed that the *H. illucens* film contributed to the increased growth of *Clostridia* spp. and restricted the growth of aerobic mesophilic bacteria and some growth of fungi. However, such results were also obtained for other films, and the reason may be the limited air flow between the atmosphere and the soil [196].

## 3. Future Perspectives

*H. illucens* is an insect that continues to capture the attention of scientists. This chapter will present the potential paths of future research on *H. illucens* in various aspects of application.

The use of *H. illucens* for the production of food and feed in western countries is faced with a major problem—it must be cost effective in comparison to other established sources, e.g., soy protein or fishmeal, which could prove difficult in the short term. Environmental problems, like overfishing could force legislators to help create laws that will promote new sources of food and feed. This could be the case in the European Union in the years to come. The nutraceutical value of insect sources associated with the content of chitin, fat, and AMPs, could help the product to come onto the market despite its higher price. At least since 2016, the literature has indicated the need to automate the insect production process, which would enable the reduction of product costs, especially in the western economic environment [197]. Currently, research in this matter is carried out e.g., [198] with the use of artificial intelligence for image recognition, and automatic solutions are already being offered commercially, such as the *Hermetia* breeding InsectoCycle (https://www.insectocycle.com) or Entoprot Ltd. (https://www.entoprot.com).

Chitin and its derivative, chitosan, are very important for many industries and this importance continues to grow. To date, chitin has been obtained from marine crustaceans, however, this source may become less relevant over time as the oceans and seas become more polluted and climate change causes unfavorable degradation in the marine environment (e.g., acidification of marine waters). Consequently, the insects, including *H. illucens*, can become an important source of chitin, especially since the world production of insects is growing, and chitinous biomass in the form of insect exuviae is a post-production waste in this process. An important aspect is also the fact that chitin obtained from different sources has different properties and, therefore, can have different applications. The aforementioned factors are a strong argument for the intensification of research on chitin and derivatives obtained from *H. illucens*.

The broad antimicrobial properties of *H. illucens* constitute a very interesting research field. It is even more important in an era in which there is increasing resistance of pathogens to commonly used antibiotics, that studies on the extraction and properties of AMPs can potentially provide new drugs to fight diseases both in medicine and in the veterinary field. Immunization with various bacteria and the use of microbially contaminated feed are the most obvious methods of initiating AMP production in *H. illucens*. The use of the latest technologies, as presented by [113], is particularly impressive, as it facilitates the treatment of entire organisms (not just insects) as “gene libraries”, worked out in silico to select the most promising peptides, which can then be synthesized completely abiotically and used in research. An innovative approach that investigated the effect of isolated AMPs on plant pathogens is also very interesting [95] and opens up a completely new field of research, with potentially important future findings. It is certain that not all such substances have already been detected and isolated. Moreover, one may ask whether changing the abiotic conditions of fly farming can affect the production and secretion of AMPs and to what extent this could be possible. The feeding of the larvae causes sanitization of the substrate from certain species of pathogenic organisms, which has been reported in some substrates, like human feces. This is particularly advantageous if such larval feeding residues are to be used later as fertilizer.

There are many reasons as to why alternative fuel (biofuel) research is still very important to economies globally, to name just a few: the zero net carbon balance in the context of climate change and sustainable development, high oil prices, steadily increasing demand for fossil fuels and increasing difficulties in accessing them. The accelerating global trend to switch energy production systems to renewable sources will not make internal combustion engines disappear overnight. The change will take years, and it should be remembered that there are many countries in the world that are not ready for such a transformation for economic reasons. In addition, internal combustion engines, especially diesel engines, may be difficult to replace with other solutions, especially in industrial applications (backup generators) as well as in ultra-heavy transport (container ships, heavy mining machinery). As an alternative fuel source, insect fat meets the same pros and cons as biodiesel produced from plant sources. However, the biggest obstacle for insect biodiesel is the scale of insect production, which is constantly increasing on a global scale, however, primary research into the use of insect fat in the near future will be in the feed, food, and cosmetics sectors. However, certain important studies into *H. illucens* larva*e* fat metabolism have been carried out. Zhu et al. [116] conducted transcriptome sequencing to investigate the mechanism of crude fat accumulation and the composition of fatty acid profiles in the different stages of *H. illucens*. Enzymes involved in lipid metabolism with expression being upregulated at the earlier stages (one to four-days old larvae) of *H. illucens* larvae development (lip, LPL, CES1, UGT, GLB1) and acting during the later stages (AKR1B, ELOVL4, and HSD17B4) were identified. This study may help clarify lipid metabolism, but it may also provide a basis for research into optimizing or designing the lipid content of *H. illucens* larvae through genetic engineering methods [116].

Although *H. illucens* waste or insect biomass itself can produce biogas in promising amounts by comparison with substrates applied in biogas plants, some studies have suggested that this type of waste should be cofermented [136]. Due to its C:N ratio and high content of lipids and proteins, the waste from *H. illucens* breeding can be mixed with substrates rich in carbohydrates like corn silage, which should improve biogas production [136]. However, there is a lack of research into this area, as the use of insects for biogas production is still a new and under investigated topic. Another interesting scientific topic will be the investigation of the composition of VFAs and the rate of its release during the methane fermentation. Some research has pointed out that insect waste has a high decomposition rate and therefore, does not require a long hydraulic retention time in the bioreactor [199]. However, there is a risk of excessive acidification connected with the evolution of VFA [136], which should be investigated more in detail. The possibility of the existence of specific compounds in insects or in insect post-production waste, which may affect the methane fermentation efficiency, remains an open question. It has been proven that the addition of extracts from certain plants, e.g., goldenrod to bioreactors resulted in enhanced biomethane production [200]. It is unknown whether such enhancement could be observed in the case of extract from insect wastes. A hypothesis worthy of future testing is that insect waste in low quantities can act as an additive to accelerate the methane fermentation of common livestock. It would be important to test various known methods of intensifying biogas production into insect waste or whole insects, as the publications currently available simply focus on determining the potential of unmodified raw material.

Entomoremediation is a fairly new research area and much more should be done in order to confirm its usefulness for different environmental issues. Certain future research challenges in this field can be predicted based on much better-established phytoremediation, i.e., the use of plants for environmental clean-up. Metallophytes, i.e., plants that prefer habitats with high concentrations of selected elements, have been known for a long time and are used as bioindicators of elevated concentrations in the soil, pinpointing areas which could be of mining importance and indicating the location of metal ores. Among metallophytes, plants capable of the hyperaccumulation of some elements were discovered, even up to extremely high levels [151]. The discovery of hyperaccumulators in 1974 [201] speeds up the progress of phytoremediation. Thus, the first question should consider whether there are any hyperaccumulators among insects. The highest element concentrations were recorded for herbivore insects fed on hyperaccumulator plants, mainly Ni-dependent, however, the term hyperaccumulator has not yet been defined and used in the case of insect (i.e., from which threshold for a given element would it be considered a hyperaccumulator) [202,203,204]. However, much more interesting is the fact that herbivores could be saprophytic insects that could be fed on different waste biomasses and could hyperaccumulate elements in any of their developmental stages. Different saprophytic species should be tested in this context. The most impressive results have already been achieved in the field of entomodegradation. This process gives hope for the effective elimination of the environmental residues of veterinary antibiotics left in manure. The use of *H. illucens* larvae for manure treatment could partly contribute to the slowing down of the phenomenon of acquiring antibiotic resistance by pathogenic bacteria.

The search for a suitable biofertilizer that reduces the use of artificial fertilizers contributes to intensifying the development of diversified management and the recirculation of nutrients in the environment, which has a positive impact on the climate. Insect frass is a promising fertilizer material, but since it has its own microbial pool, the relationships, and interactions of the microorganisms with the soil and plants, is very interesting for in depth studies. For instance, in what conditions (diet or parameters of breeding) can pathogenic organisms survive in the frass and be delivered to the soil? Some bacteria known as plant growth promoting bacteria (PGPR) can produce phytohormone-like substances, which promote plant growth [205]. It is possible, however, it has yet to be tested, whether insect frass can contain any substances directly influencing plant growth. If this is true, insect frass could be viewed as a functional fertilizer. A noteworthy issue concerns the chitinous residues, which are left in the frass as fragments of insect exoskeletons. In in vitro plant cultures, chitin and chitosan are well known plant elicitors, i.e., compounds which stimulate the stress resistance of plants [206]. The presence of chitin and chitin fragments cut by microbial chitinases could be a positive factor, influencing plant health during the use of insect frass as fertilizers. It is also important to optimize and standardize substrates for *H. illucens* larvae, to obtain N, P, K rich fertilizer in known ratios. Frass stability and the need for its maturation, as in the case of composts, are additional interesting issues. Does frass already contain any humic substances, which are important for soil health or does the maturation of the frass cause them to develop, making frass a more stable and valuable fertilizer? These are questions not yet answered in the literature.

## Figures and Tables

**Table 1:** Compilation of animals as test objects and *H. illucens* as a form of feeding.

Animal	Form of *H. illucens*	Effects	References
Positive influence			
Laying hens	Soybean meal and soybean oil + *H. illucens* pre-pupae	Increased egg weight by 1.1 times, increased SCFA concentration by 1.3 times	[32]
Laying hens	Corn-soybean meal + 25% of replaced protein by partially defatted *H. illucens*	Despite the reduced length of the intestinal villi, increased the amount of volatile fatty acids by 1.1 times and the amount of butyrate by 1.2 times in intestines	[33]
Hen broilers	Chicken feed + 5% or 10% of live *H. illucens* larvae	Decreased timidity of hens, increased activity of hens	[34]
Atlantic salmon (*Salmo salar*)	Corn protein, soybean meal + (200 g·kg^−1^) *H. illucens* meal	No significant differences to control	[36]
European seabass (*Dicentrarchus labrax*)	Fishmeal + 50% dried *H. illucens* larvae meal	No significant differences to control	[38]
Finishing pigs	Corn, wheat bran and soybean meal + 4% dried and crushed *H. illucens* prepupea	Decreased expression of pro-inflammatory cytokines and concentrations of total amines and phenol, increased expression of anti-inflammatory cytokines, intestinal barrier genes and concentrations of short-chain fatty acids (SCFA) and butyrate (prebiotic effect)	[39]
Weanling piglets	Fishmeal + 2% full-fat *H. illucens* larvae meal	Increased lactate in *illeum* by 1.6 times, in *cecum* by 2.2 times, and SCFA by 1.2 times in *illeum* and by 1.1 in *cecum* (probiotic effect), increased anti-inflammatory protein IL-10 by 1.3 times, decreased pro-inflammatory protein TNF-α by 1.3 times	[40]
Beagle dogs	Grain-based diet + 2% defatted *H. illucens* larvae meal	Improved dry matter digestibility by 1.1 times, decreased TNF-α levels by 1.8 times (anti-inflammatory effect), increased glutathione peroxidase levels by 1.23 times (antioxidant effect)	[41]
Rabbits	Rabbits feed + 1.5% *H. illucens* fat	Inhibition of the growth of the pathogens *Pasteurella multocida* by 3.2 times, *Yersinia enterocolitica* by 2.5 times, *Listeria monocytogenes* by 2.1 times	[43]
Muscovy ducklings (*Cairina moschata domestica*)	9% partially defatted *H. illucens* meal	Decrease in uric acid by 1.2 times and creatinine by 1.2 times (improved kidney function), increase in serum iron Fe by 1.3 times	[45]
African catfish (*Clarias gariepinus*)	Fishmeal + 50% partially defatted *H. illucens* larvae meal	Increase in body weight by 1.5 times	[47]
Rainbow trout (*Oncorhynchus mykiss*)	Control diet (wheat gluten, soybean meal and hemoglobin) + 15% *H. illucens* larvae meal	Increase in the number of beneficial *Lactobacillus* and *Bacillus* bacteria, reduction in *Aeromonas* pathogens in fish gut	[48]
Female turkeys	Soybean-maize enriched with 50 g/kg *H. illucens* larvae fat (50% and 100%)	Improved intestinal digestibility of the ether extract Increase in lipase activity Reduction of *Bacteroides-Prevotella* clusters	[50]
Negative influence			
Meagre (*Argyrosomus regius*)	Partially defatted *H. illucens* + fishmeal	Weight loss, decrease in protein efficiency	[37]
Atlantic salmon (*Salmo salar*)	Control diet with full-fat *H. illucens* larvae meal, substituting 12.5% content of protein and control diet with full-fat *H. illucens* larvae paste, substituting 6.7% of protein	Decrease in protein and lipid efficiency and protein efficiency index, decrease in phosphorus retention	[49]

**Table 2:** Total chitin content (%) in different developmental stages of *H. illucens* and puparia.

Extraction Methods	Insect Material	Chitin Content (%)	Crystalline Index (%)	References
Demineralization: 1 M HCl (1 h), deproteinization: 1 M NaOH (80 °C, 24 h), depigmentation: 1% KMnO_4_	puparium	n.d.	35.0	[64]
	adults	n.d.	24.9	
Demineralization: 2 M HCl (55 °C, 1 h, 200 rpm·min^−1^), deproteinization: 2 M NaOH (50 °C, 18 h, 200 rpm·min^−1^), depigmentation: 3.6% HCl (0.5 h), 10-fold diluted NaClO (80 °C, 4 h, 200 rpm·min^−1^)	larvae	3.6	33.1	[65]
	prepupae	3.1	35.1	
	puparium	14.1	68.4	
	adults	2.9	87.92	
Demineralization: 1 M HCl (1:10 (*m*:*v*), room temp., 1 h), deproteinization: 1 M NaOH (1:25, 80 °C, 1 h), depigmentation: 12-fold repetition of the deproteinization process	larvae	9.5	~88	[67]
	prepupae	9.1	~95	
	pupae	10.3	~93	
	larvae shedding	31.1	~90	
	puparium	23.8	~94	
	adults	5.6	~89	
Demineralization: 1 M HCl (100 °C, 0.5 h), deproteinization: 1 M NaOH (24 h)	puparium	25.4	74.1	[68]
	flakes after oil extraction	20.7	61.1	
	adults	7.8	77.8	
Acid detergent fiber—acid detergent lignin	puparium	21.2	70.8	
	flakes after oil extraction	26.8	50.0	
	adults	7.9	39.0	
Demineralization: 1 M HCl, 22 °C, 1 h, deproteinization: 1 M NaOH, 80 °C, 24 h, depigmentation: 9% H_2_O_2_, 80 °C, 2.5 h	puparium	7.0	60.0	[69]
Demineralization: 0.5 M formic acid (1:10 (*m*:*v*)), 1 h, room temperature, deproteinization: 2 M NaOH (1:10 (*m*:*v*)), 2 h, 80 °C	larvae	13.0	74.0	[70]
	puparium	31.0	78.0	
	adults	9.0	79.0	
Demineralization: 0.5 M formic acid (1:10 (*m*:*v*)), 1 h, room temperature, deproteinization: 2 M NaOH (1:10 (*m*:*v*)), 2 h, 80 °C, depigmentation: 5% H_2_O_2_, (1:20–30), 30–60 min, 90 °C	larvae	10.0	77.0	
	puparium	23.0	80.0	
	adults	6.0	86.0	

n.d.—no data.

## Data Availability

Not applicable.
